# Middle-ear cholesteatoma co-existing with labyrinthine fistula and vestibular schwannoma

**DOI:** 10.1007/s00405-020-05796-0

**Published:** 2020-01-23

**Authors:** Aleksander Zwierz, K. Masna, P. Burduk

**Affiliations:** 1 Department of Otolaryngology, Oncology and Oral and Maxillofacial Surgery, Faculty of Health Sciences, Collegium Medicum in Bydgoszcz, Nicolaus Copernicus University, Ujejeskiego Street 75, 85-168 Bydgoszcz, Poland; 2 Departament of Phoniatry and Audiology. Faculty of Health Sciences, Collegium Medicum in Bydgoszcz, Nicolaus Copernicus University, Bydgoszcz, Poland

**Keywords:** Vestibular schwannoma, Cholesteatoma, Sensorineural hearing loss, Vertigo, Tinnitus

## Abstract

**Background:**

Many conditions, among them vestibular schwannoma and middle ear cholesteatoma with lateral semicircular canal destruction, may be associated with asymmetrical sensorineural hearing loss (SNHL) and vertigo. However, the probability that these two distinct disease entities causing the same symptoms occur in a single patient is very low, approximately 1 per 28 billion per 1 year.

**Methods:**

We present the case of a 40-year-old male admitted to our clinic because of chronic middle ear inflammation with concomitant tinnitus vertigo, and deafness in the right ear. The patient was diagnosed with lateral semicircular canal fistula caused by middle-ear cholesteatoma and concomitant vestibular schwannoma. Canal wall-down surgery was carried out to remove the cholesteatoma, followed by gamma knife radiosurgery for the vestibular schwannoma.

**Results:**

Vertigo and tinnitus resolved within 3 days after the ear surgery, and gamma knife treatment resulted in the complete involution of the vestibular schwannoma. The patient presented with completely dry middle-ear cavity and no recurrence of the cholesteatoma was observed during a 3-year follow-up.

**Conclusion:**

As the hereby reported condition is very rare, the results cannot be compared with any similar report published previously. Nevertheless, based on the outcome, the treatment strategy seems to be both reasonable and effective.

## Introduction

Asymmetrical sensorineural hearing loss (SNHL) or deafness and vertigo may have many causes, among them vestibular schwannoma (VS) and chronic otitis media with cholesteatoma formation and destruction of the inner ear structures, such as lateral, superior and posterior semicircular canals and promontory [1]. The incidence of cholesteatoma in adults is reported at 9.2 per 100,000 per year [2], and in 2.7–12% of the cases, this condition may eventually lead to the development of labyrinthine fistula [1, 3] Nearly 90% of labyrinthine fistulas caused by cholesteatoma are located in the lateral semicircular canal, and profound SNHL is noted in 15% of the patients [1]. According to various sources, the incidence of VS varies from 12 to 19.4 per 1 million per year [4, 5]. Considering the low incidence of either cholesteatoma or VS, the probability that these two different entities occur in a single patient is very low, approximately 1 per 28 billion per year, which corresponds to one person worldwide every 4 years. To the best of our knowledge, only one case of cholesteatoma and VS occurring in the same patient has been described thus far, albeit not in the same ear. Below, we present the case of a patient with same-side VS and middle-ear cholesteatoma complicated by labyrinthine fistula; we put special emphasis on the available therapeutic options and treatment outcomes.

## Case report

**Fig. 1:**
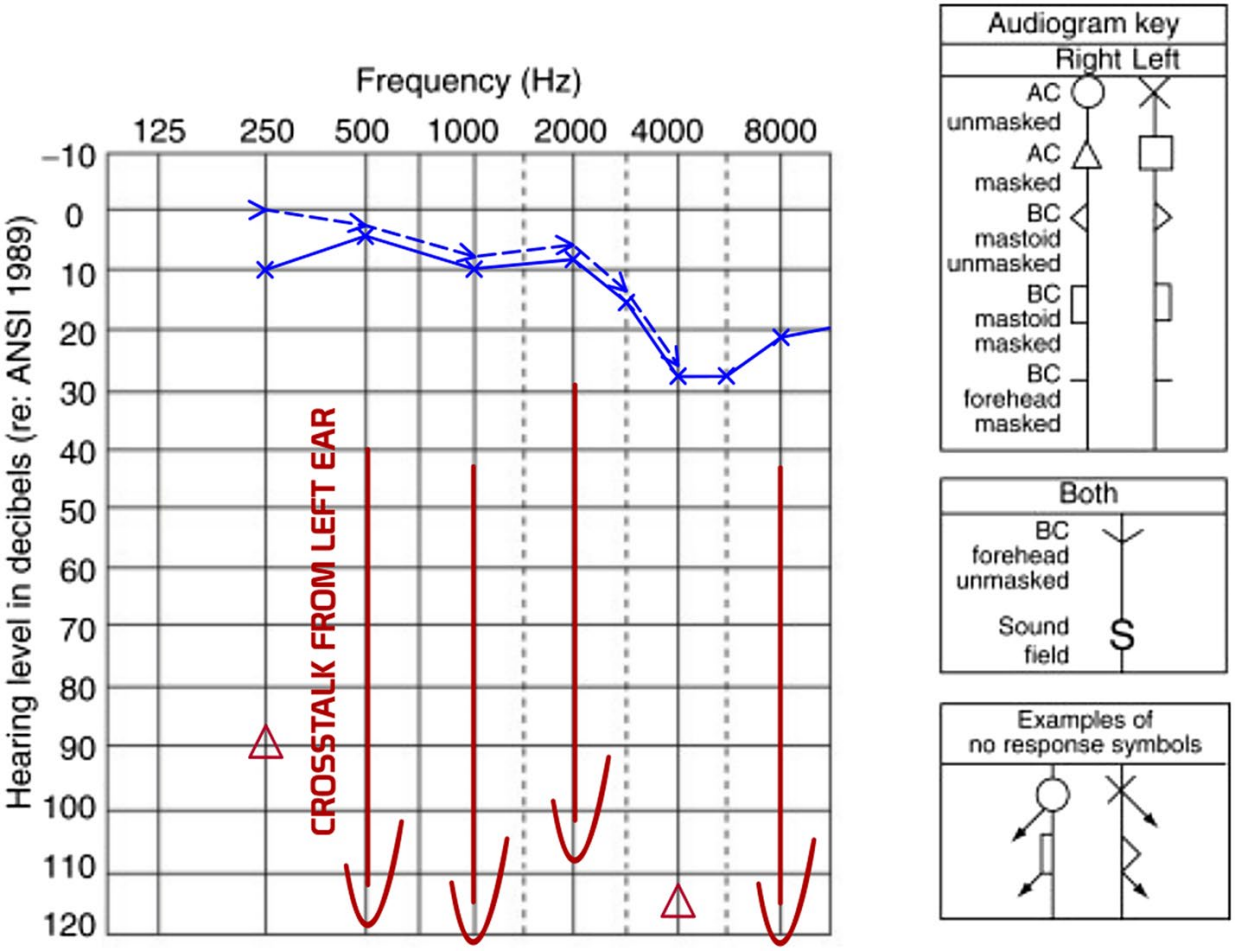
Pure tone audiometry. Completely deaf right ear

A 40-year-old male was referred to our ENT department with chronic otorrhea that started 4 years earlier, as well as with vertigo and tinnitus that began 6 months before the admission. The patient had a long-term history of right-ear hypoacusis which, according to his mother, started at 11 years of age. The otoscopic examination demonstrated subtotal perforation of the tympanic membrane and keratin masses arising from the attic. Lateralization to the left ear was observed during the Weber test, and the patient tested positively for the Hennebert's sign. No signs of facial nerve dysfunction and taste disorders were noted. Tonal audiometry showed complete hearing loss in the right ear (Fig. 1), with no response to otoacoustic emission and auditory brainstem response (ABR) V wave of 90 dB and interlatency of 9 ms.

**Fig. 2:**
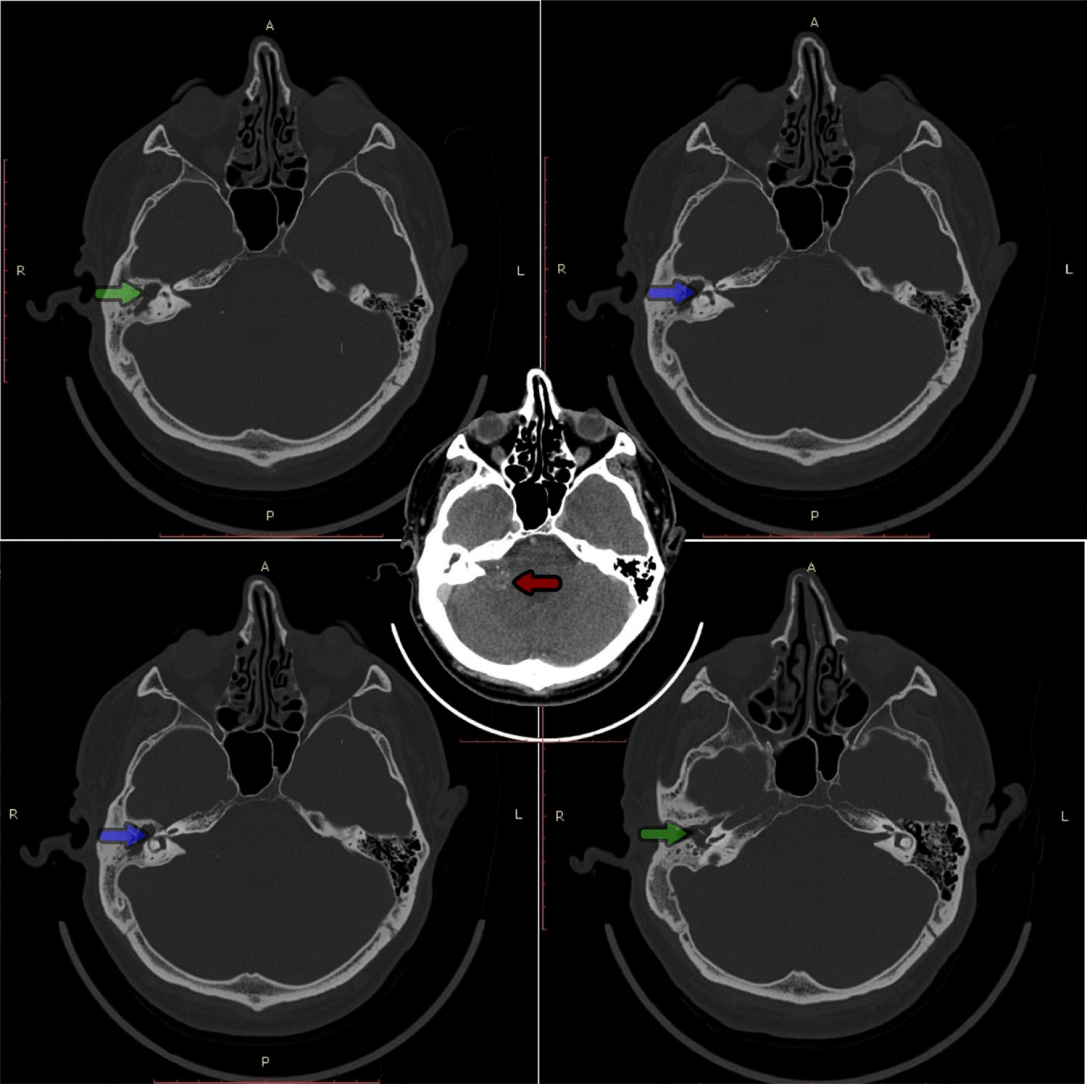
HRCT scan of the ears (before surgery). On the centre scan pathology of the middle fossa—right CP-angle tumor—red arrow. On the surrounding scans—pathology of the middle ear. Cholesteatoma of the tympanic cavity and mastoid (green arrow), lateral semicircular canal fistula (blue arrow)

**Fig. 3:**
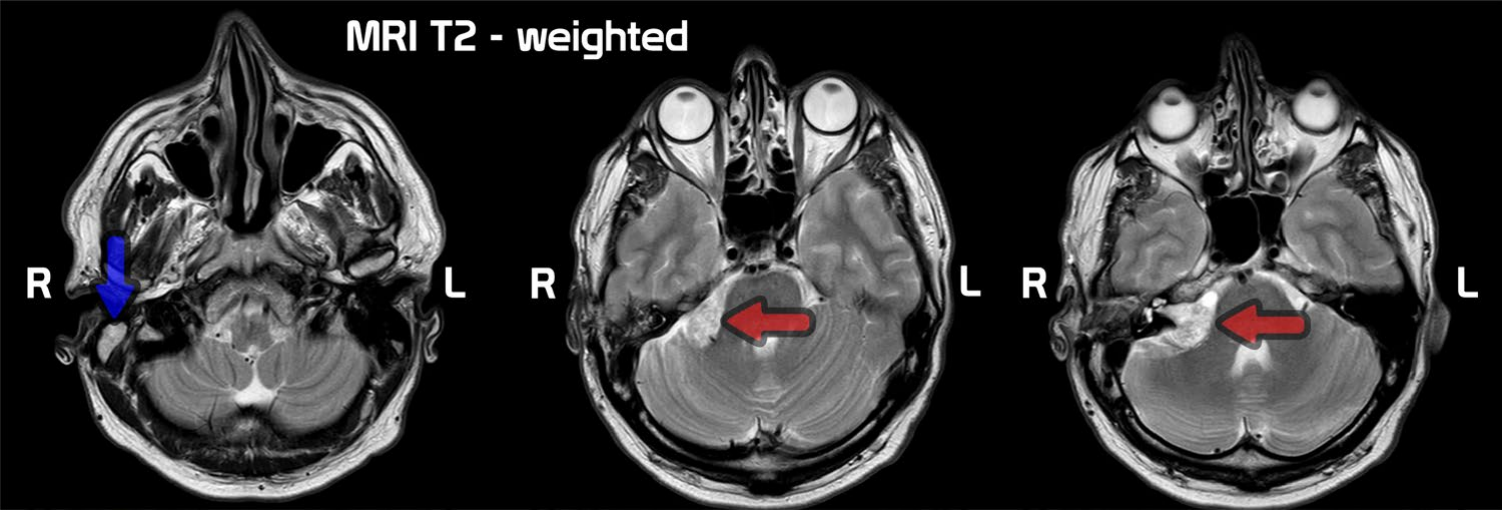
T2 MRI of the cerebellopontine angle (before surgery). Cholesteatoma of the tympanic cavity and mastoid (blue arrow). Right CP-angle tumor (red arrow)

Computed tomography (CT) demonstrated a solid-cystic mass (17 × 18 × 20 mm) with calcification foci in the right cerebellopontine angle. Moreover, complete opacification of mastoid cells was observed, along with a mass in the tympanic cavity, causing destruction to the lateral semicircular canal (Fig. 2). Magnetic resonance imaging (MRI) confirmed the presence of the characteristic signs of VS in the cerebellopontine angle and cholesteatoma in the right ear (Fig. 3).

The patient underwent canal wall-down surgery with removal of the cholesteatoma. The procedure also included reconstruction of the tympanic membrane and obliteration of the mastoid cavity. Vertigo and tinnitus resolved within several days after the ear surgery.

**Fig. 4:**
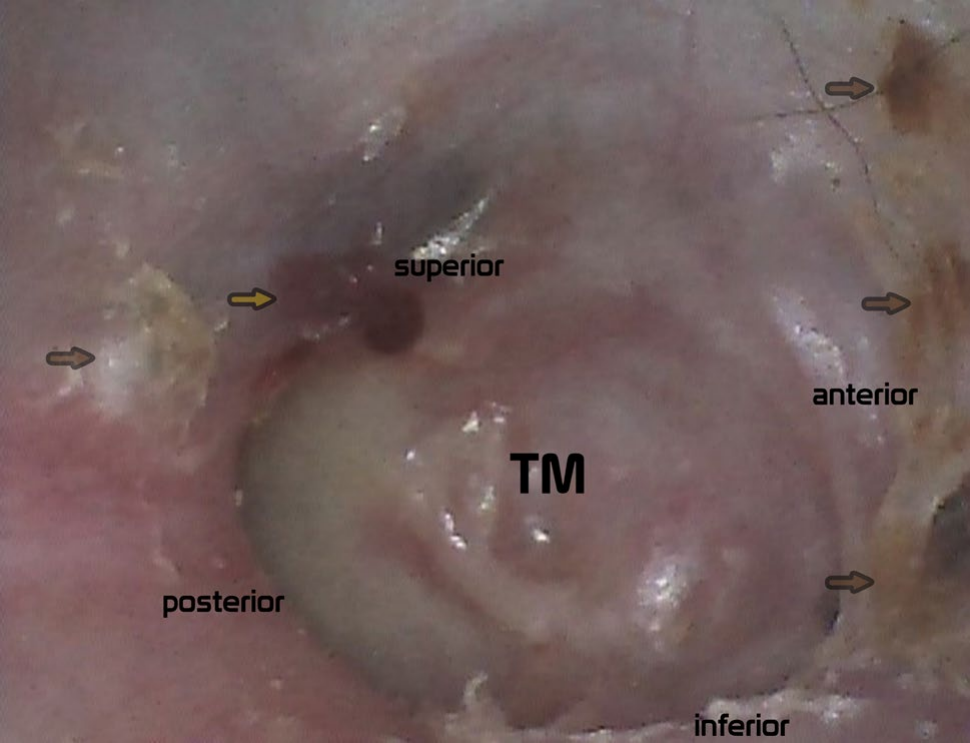
Otoscopy (24 month post surgery). Reconstructed tympanic membraine (TM). Small hematoma—artifact after ear cleaning (yellow arrow), remains of keratonized surface epithelium (brown arrow)

When the ear cavity healed completely, the patient underwent gamma knife radiosurgery. Control MRI with Gadoteridol contrast agent demonstrated involution of the VS and reduction of the post-contrast enhancement. On otoscopic examination, the ear was completely dry, with no signs of cholesteatoma recurrence (Fig. 4). Currently, the only ailment reported by the patient is intermittent tinnitus occurring while he leans forward and lasting no longer than a few seconds.

## Discussion

To the best of our knowledge, this is the first published report on lateral semicircular canal fistula caused by middle-ear cholesteatoma, co-existing with VS in the same ear. The probability that a single patient developed these two conditions that may both result in deafness and vertigo is very low. It is unclear whether deafness and vertigo present in our patient were associated with VS or chronic middle-ear inflammation and resultant lateral semicircular canal fistula, as these symptoms are characteristic for the progress of both conditions mentioned above. Currently, the treatment of choice in chronic middle-ear inflammation with cholesteatoma and semicircular canal fistula development may be canal wall-down procedure with removal of the middle-ear pathologies. Cholesteatoma matrix should be removed completely.

Both our experiences and published data suggest that vertigo may resolve as a consequence of VS treatment. According to literature, up to 80% of patients subjected to gamma knife radiosurgery no longer presented with vertigo [6, 7]. However, early and late vertigo remission rates after subtotal surgical resections of large VS are no greater than 30% and 14%, respectively [8]. Since our patient no longer presented with vertigo and tinnitus after the middle-ear surgery, these ailments were likely associated with the chronic ear inflammation and semicircular canal fistula. SNHL in patients with chronic otitis media and cholesteatoma may not only be caused by the migration of inflammatory mediators or toxins into the inner ear fluid from the inflammation site [1], but can also be a consequence of VS growth. Identification of the hearing loss etiology in such cases can be challenging, and improvement of hearing function is unlikely.

We carefully analyzed available treatment options in the hereby presented case. Three (four?) different strategies of VS management can be used: microsurgical resection, fractionated radiotherapy, stereotactic radiosurgery or "watch and scan" approach, i.e., observation with serial imaging to detect potential growth of the tumor [9]. The most popular methods for surgical resection are translabyrinthine, middle-fossa and retrosigmoid approaches. Each of these methods poses a 0.5% mortality risk [10]. However, non-surgical strategies, such as gamma knife stereotactic radiosurgery or linear acceleration of electrons (LINAC) are gaining growing popularity nowadays in the treatment of medium-sized tumors with diameters between 1 cm and 2–2.5 cm [11]. The "watch and scan" strategy applies to smaller tumors, with no more than 1 cm in diameter [10], as well as to older patients, > 65 years of age. The decision whether to perform the surgical resection is determined not merely by the tumor size, but also patient's age, surgeon's experience and local guidelines [9], and the physician should always discuss all these factors with the patient.

In the hereby presented case, persistent otorrhea made the patient non-eligible for the most commonly performed type of surgical procedure, translabyrinthine resection [9]. Furthermore, the tumor was very big for the hearing–sparing procedure, retrolabyrinthine resection, and the patient already presented with hearing loss. Hence, the available treatment options included middle-fossa approach, retrosigmoid resection, gamma knife radiosurgery and watchful waiting. Recently, a trend to the more frequent application of radiation therapy than surgical resection is observed, with the former used in twice as many patients as the latter [12]. Another argument for the use of radiation therapy is that most neurosurgeons report problems with intraoperative identification of the facial nerve, and hence, prefer incomplete resection of the tumor to avoid an iatrogenic facial palsy [9]. According to literature, the quality of life in patients treated with radiotherapy is satisfactory, and failure rates with progressive growth of the tumor seem to be similar as the overall recurrence rates after the surgical treatment [12, 13]. While complete surgical removal of a small VS can preserve hearing and facial nerve function, it does not eliminate the risk of tinnitus and vertigo [11]; according to Lund–Johansen [11], persistent vertigo has the most detrimental effect on the quality of life in patients after VS resection.

As our patient did not consent for neurosurgery, gamma knife radiosurgery was the only radical treatment option. In line with current recommendations, the stereotactic radiotherapy was preceded by the treatment of chronic ear inflammation. To shorten the recovery time and to facilitate the introduction of gamma knife therapy, we performed canal wall-down procedure as a one-stage cholesteatoma surgery. We have meticulously removed the whole epithelial lining, covered the fistula with temporalis fascia and obliterated the ear cavity with the temporalis muscle to decrease its volume. Such an approach to labyrinthine fistula has been previously recommended by Sagar et al. [14]. No recurrence of cholesteatoma was observed during a 3-year follow-up period, and the patient no longer reported vertigo. Furthermore, complete involution of VS was achieved with gamma knife therapy.

## Conclusion

As the hereby reported condition is very rare, our experiences cannot be compared with any similar report published previously. Nevertheless, based on the outcome, the treatment strategy applied in our patient seems to be both reasonable and effective.

## References

[1] Copeland BJ, Buchman CA (2003). Management of labyrinthine fistulae in chronic ear surgery. Am J Otolaryngol.

[2] Olszewska E, Wagner M, Bernal-Sprekelsen M (2004). Etiopathogenesis of cholesteatoma. Eur Arch Otorhinolaryngol.

[3] Letícia PSR, Canali I, Teixeira A, Silva MN, Selaimen F, da Costa SS (2010). Cholesteatoma labyrinthine fistula: prevalence and impact. Neurosurgery.

[4] Stangerup SE, Tos M, Thomsen J, Caye-Thomasen P (2002). True incidence of vestibular schwannoma?. J Laryngol Otol.

[5] Babu R, Sharma R, Bagley JH, Hatef J, Friedman AH, Adamson C (2013). Vestibular schwannomas in the modern era: epidemiology, treatment trends, and disparities in management: clinical article. J Neurosurg.

[6] Tuleasca C, George M, Schiappacasse L, Patin D, Fenu J, Maire R, Levivier M (2019). Gamma Knife radiosurgery for intravestibular and intracochlear schwannomas. Acta Neurochir.

[7] Deberge S (2018). Quality of life in the management of small vestibular schwannomas: observation, radiotherapy and microsurgery. Clin Otolaryngol.

[8] MacKenzie R (2018). The difficulty of predicting clinical outcome after intended submaximal resection of large vestibular Schwannomas. J Clin Neurosci.

[9] Hentschel M, Rovers M, Markodimitraki L, Steens S, Kunst H (2019). An international comparison of diagnostic and management strategies for vestibular schwannoma. Eur Arch Oto-Rhino-Laryngol.

[10] Bashjawish B, Kılıç S, Baredes S, Eloy JA, Liu JK, Ying Y-LM (2019). Changing trends in management of vestibular schwannoma: a national cancer database study. Laryngoscope.

[11] Lund-Johansen M (2018). Treatment of small and medium-sized vestibular schwannoma—a need for better evidenc. Acta Neurochir.

[12] Myrseth E, Moller P, Pedersen PH, Lund-Johansen M (2009). Vestibular schwannoma: surgery or gamma knife radiosurgery? A prospective, nonrandomized study. Neurosurgery.

[13] Regis J, Pellet W, Delsanti C (2013). Functional outcome after gamma knife surgery or microsurgery for vestibular schwannomas. J Neurosurg.

[14] Sagar P, Devaraja K, Kumar R, Bolus S, Sharma S (2017). Cholesteatoma induced labyrinthine fistula: is aggressiveness in removing disease justified?. Indian J Otolaryngol Head Neck Surg.

